# Automated Nuchal Translucency analysis for early prenatal risk stratification

**DOI:** 10.3389/frai.2026.1914775

**Published:** 2026-10-01

**Authors:** Smita S. Pawar, Mangesh D. Nikose, Prakash G. Burade

**Affiliations:** 1 Electrical and Electronics Department, Sandip University, Nashik, India; 2 DYPIU, Akurdi, India

**Keywords:** ACM, EMSSTGAN, FRANet, MARN, NT, U-net

## Abstract

Nuchal Translucency (NT) is a measurement taken from ultrasound images described as the gathering of fluid behind the fetus's neck, during 11–13 weeks. Increased Nuchal Translucency can lead to high risk of chromosomal abnormalities like Down Syndrome. The existing AI model may struggle to detect the subtle difference due to low resolution fetal movement, which can lead to errors in measurements and diagnosis. In addition, traditional NT measurement methods rely heavily on manual caliper placement, making the process operator-dependent and reducing consistency and automation. To address these challenges, this paper proposes an Enhanced Multi-Scale Spatial Temporal Graph Attention Network (EMSSTGAN) to predict the risk of chromosomal abnormalities in NT. Initially, the input images of NT are pre-processed using feature refinement attention network (FRANet) for denoising and Multi-Scale Attention Retinex Network (MARN) for contrast enhancement. NT detection is then performed by localizing the nuchal translucency region using bounding box annotations, followed by U-Net-based segmentation for precise extraction of the NT area. The Active Contour Model (ACM) is subsequently applied to measure the NT thickness based on boundary geometry. The thickness when compared with threshold values is used to identify the risk of chromosomal abnormalities as low, medium and high. The proposed model achieves an accuracy of 98.15%, an error of 1.85%, and a FDR of 2.44%, respectively.

## 1 Introduction

Nuchal translucency (NT) is the fluid-filled space beneath the skin at the back of the fetal neck, visible on grayscale ultrasound images between two echogenic regions (Zhao et al., 2025). An increased NT measurement during the first trimester is associated with a higher likelihood of chromosomal abnormalities, particularly Down syndrome (DS), in fetuses (Su et al., 2021). NT achieves its maximum thickness and is best measured between 11 and 14 weeks of gestation (Pawar and Nikose, 2025). During this period, ultrasound examination also helps assess important fetal anatomical features, and additional abnormalities detected during NT screening may indicate a higher risk of structural or genetic disorders. The cutoff value of NT refers to the threshold thickness used to classify fetuses into normal or high-risk groups for chromosomal abnormalities (Xu et al., 2023). Other complications such as miscarriage, stillbirth, preeclampsia, gestational diabetes mellitus, premature delivery, fetal growth restriction, and macrosomia can also be identified during this stage (Wang et al., 2023). Various traditional methods such as the Sobel Operator, semi-automatic methods, and non-linear anisotropic filter techniques have been used to measure NT thickness. However, these methods often face challenges due to the low-contrast boundaries of the NT region and variations in image intensity, which affect accurate detection.

Since these methods rely heavily on manual placement of calipers and initial inputs by sonographers, they increase inter- and intra-observer variability and reduce repeatability. In addition, careful adjustment of parameters such as scale, iteration count, and diffusion coefficients is required, where incorrect settings may lead to insufficient smoothing or excessive blurring (Pawar et al., 2025). To overcome these limitations of traditional methods, Artificial Intelligence (AI) can be employed for the automatic detection of NT.

The use of Artificial Intelligence and deep learning has improved diagnosis analysis in medical imaging (Ługowski et al., 2026). Artificial intelligence has significantly improved NT diagnosis by enhancing imaging techniques and prediction models (Elger et al., 2025). Machine Learning (ML) models such as K-Nearest Neighbors (KNN), Multi-layer Perceptron (MLP), Minimal Learning Machine (MLM), and Extreme Learning Machine (ELM) have been applied for NT analysis (Yasrab et al., 2022); however, they suffer from limitations such as vanishing gradient problems during training, the need for extensive annotation for precise segmentation, misclassifications due to fetal head posture variations, and difficulties in handling unclear head-NT interactions (Zhao et al., 2025; Hynek et al., 2021). Furthermore, these models are computationally expensive when dealing with large NT datasets, and real-time analysis becomes challenging due to the time-consuming calculation of distances between new and existing data points (Mavaluru et al., 2024).

To overcome these ML limitations, Deep learning techniques are used that recognizes intricate patterns and makes predictions from vast amount of data (Karadağ et al., 2021). These models use various techniques, including Long Short-Term Memory (LSTM), Deep Neural Network (DNN), Convolutional Neural Network (CNN), and Recurrent Neural Network (RNN) (Kunanukulwatana et al., 2025). Although these techniques perform better than conventional ML approaches, they still face challenges such as slow training speed, difficulty in handling sequential data, high computational complexity, overfitting, and poor generalization to unseen data (Nasri et al., 2024; Qian et al., 2023). Additionally, the lack of transparency and difficulty in explaining the decision-making process remain major concerns in these models (Hu et al., 2022). To address these challenges, an enhanced Multi-Scale Spatial Temporal Graph Attention Network (EMSSTGAN) is proposed for automated NT detection and chromosomal abnormality risk prediction.

Major contribution of the work is given below

- Feature Refinement Attention Network (FRANet) technique is used for image denoising while Multi-Scale Attention Retinex Network (MARN) technique is used to enhance the contrast
- NT detection is performed by identifying and localizing the nuchal translucency region in fetal ultrasound images using bounding boxes annotations
- NT region is segmented using a U-Net model, and an Active Contour Method (ACM) is applied to accurately delineate its boundaries for precise thickness measurement.
- An Enhanced MSSTGAN is used to classify the risk of nuchal by analyzing the measured NT thickness and categorizing cases into low, medium, and high-risk groups.

## 2 Proposed methodology

Traditional NT measurement methods are mainly based on manual caliper placement by sonographers, which makes the process highly operator-dependent. In addition, low image contrast, fetal movement, and unclear NT boundaries can lead to inaccurate measurements. To overcome these several issues, automated techniques are required to improve precision, consistency, and reliability in NT detection. This study proposes image enhancement, automatic NT measurement, and risk categorization from ultrasound images for improved prenatal screening. Figure 1 shows the workflow of automated NT detection and Risk classification. Initially, the input nuchal translucency (NT) scans are obtained from fetal ultrasound images collected from the Mendeley dataset. These images are first preprocessed using the Feature Refinement Attention Network (FRANet) to remove unwanted noise and improve feature quality, followed by the Multi-Scale Attention Retinex Network (MARN) for contrast enhancement and overall image quality improvement. After preprocessing, the NT region is localized using bounding box annotations, and segmentation is performed using the U-Net architecture to accurately extract the NT region from the image boundaries. The segmented output is then provided to the Active Contour Model (ACM) for thickness detection, where the NT thickness is measured based on the precise boundary geometry of the segmented region. In classification phase, enhanced Multi-Scale Spatial Temporal Graph Attention Network (MSSTGAN) to classify the risk of chromosomal abnormalities. For enhancing the MSSTGAN, the technique of transformer attention is replaced by transformer-based multi-head attention, where transformer-based multi-head attention model can understand how data can change overtime and it gives accurate predictions. According to the specifications, the output will be shown as Low, Medium, and High.

**Figure 1:**
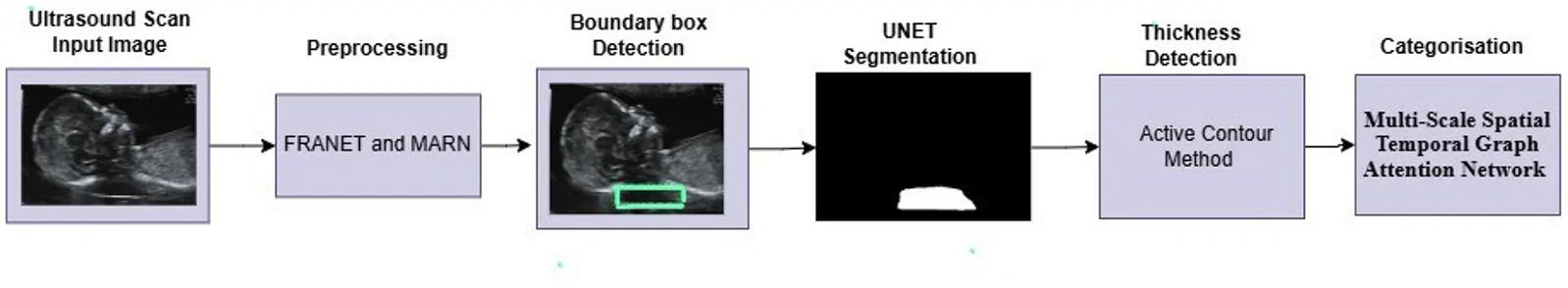
Architecture of automated nuchal translucency detection and risk classification.

### 2.1 Dataset description

The Shenzhen People's Hospital provided the dataset, which consists of 1,528 2D sagittal-view ultrasound images of 1,519 female patients (Cui and Dong, 2022). Each image is accompanied by bounding box annotations that define the nuchal translucency region (NT) in terms of minimum and maximum width and height coordinates.


$$\left(w_{\min},h_{\min},w_{\max},h_{\max}\right)$$

Following preprocessing and augmentation, the primary dataset was divided into training and testing subsets using an 80:20 split. In addition, 156 independent ultrasound images from the Longhua branch of Shenzhen People's Hospital were incorporated into the evaluation data. Overall, a total of 794 samples were used for evaluation.

### 2.2 Preprocessing

The dataset exhibits variations in image quality, including noise, low contrast, and occasional missing or null values, necessitating preprocessing steps such as denoising, contrast enhancement, and normalization to improve data consistency and reliability. In this model, the Feature Refinement Attention Network (FRANet) and Multi-Scale Attention Retinex Network (MARN) are employed for denoising and contrast enhancement. FRANet takes noisy images as input, removes noise and improves the important features (Liu et al., 2025). Figure 2 illustrates the preprocessing of ultrasound images. Images on left side are the original scan while those on the right side are the preprocessed scan after denoising and contrast enhancement.

**Figure 2:**
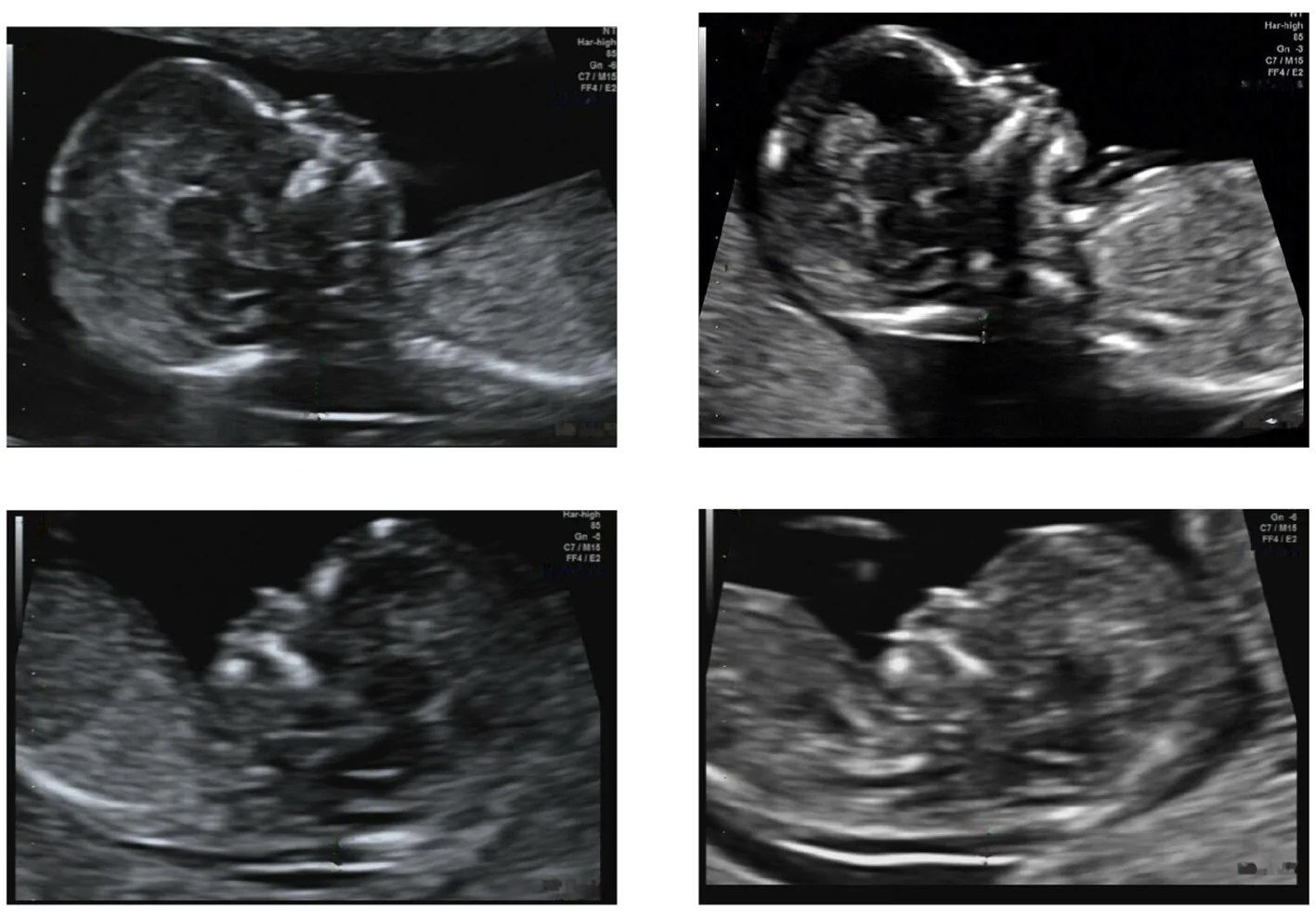
Original images and preprocessed images.

#### 2.2.1 Feature refinement attention network

The noisy input image is first refined using the Feature Refinement Attention Network (FRANet), which accelerates network training and extracts more discriminative features. The noisy image is provided as input, and the refined feature representation is obtained through the feature refinement network, which can be expressed as shown in Equation 1:


$$\begin{array}{l} f_{fr}=F_{FR}\left(X_{N}\right) \end{array}$$ (1)

where *F*_*FR*_ denotes the feature refinement function, *X*_*N*_ represents the noisy input image, and *f*_*fr*_ corresponds to the refined feature representation.

The refined feature is subsequently fed into the feature attention encoder–decoder network. This network extracts deep feature representations from the image, which can be expressed as shown in Equation 2:


$$\begin{array}{l} f_{\text{faed}}=F_{\text{FAED}}\left(f_{fr}\right) \end{array}$$ (2)

where *F*_*FAED*_ denotes the Feature Attention Encoder–Decoder function and *f*_*faed*_ represents the extracted deep features.

To obtain the final network output, FRANet reconstructs the enhanced image from the extracted deep features, as shown in Equation 3


$$\begin{array}{l} Y_{C}=F_{\text{FRANet}}\left(X_{N}\right)=X_{N}-F_{C}\left(f_{\text{faed}}\right) \end{array}$$ (3)

where *F*_*FRANet*_ represents the mapping function of FRANet, *Y*_*C*_ denotes the reconstructed output image, and *F*_*C*_ represents the convolution operation. FRN is primarily employed for feature refinement of noisy input images, ensuring that image information is preserved as much as possible during the denoising process. Initially, three successive convolutional layers are applied to extract preliminary features, which can be expressed as shown in Equation 4:


$$\begin{array}{l} f_{origin}=F_{C}\left(R\left(F_{C}\left(F_{C}\left(X_{N}\right)\right)\right)\right) \end{array}$$ (4)

where *R* denotes the ReLU activation function and *F*_*C*_ represents the convolution operation. Subsequently, channel attention and pixel attention mechanisms are incorporated into the feature refinement network after the channel split and shuffle operations. These attention modules enable the network to emphasize informative features while suppressing irrelevant information, thereby enhancing detailed feature extraction. The refined feature representation is computed as shown in Equation 5:


$$\begin{array}{l} f_{fr}=H_{PA}\left(H_{CA}\left(F_{C}\left(R\left(F_{C}\left(f_{CSS}\right)\right)+f_{CSS}\right)\right)\right)+f_{CSS} \end{array}$$ (5)

where *H*_*PA*_ and *H*_*CA*_ denote the pixel attention and channel attention operations, respectively. The function *R* represents the ReLU activation function, *f*_*CSS*_ denotes the feature map obtained after the channel split and shuffle operations, and *f*_*fr*_ represents the final refined feature output.

#### 2.2.2 Multi-scale attention retinex network

The Multi-Scale Attention Retinex Network (MARN) is designed to estimate a comprehensive inverse illumination map by incorporating color and detail information for low-light image enhancement (Zhang and Wang, 2021). Retinex theory has been widely employed to improve low-light images by decomposing an image into reflectance and illumination components. It can be expressed as shown in Equation 6:


$$\begin{array}{l} S\left(x,y\right)=R\left(x,y\right)*I\left(x,y\right) \end{array}$$ (6)

where * denotes pixel-wise multiplication, (*x, y*) represents the pixel location, *I* denotes the illumination component, *R* represents the reflectance component, and *S* denotes the observed image.

The objective of MARN is to provide the network with prior knowledge regarding underexposed regions, enabling it to focus on poorly illuminated areas while preserving well-exposed regions. The illumination-attention map is formulated as shown in Equation 7:


$$\begin{array}{l} IA=\underset{c}{\min}\left(\frac{\underset{c}{\max}\left(S\right)-S}{\underset{c}{\max}\left(S\right)-\underset{c}{\min}\left(S\right)}\right) \end{array}$$ (7)

where $\underset{c}{\max}\left(\cdot \right)$ and $\underset{c}{\min}\left(\cdot \right)$ denote the maximum and minimum intensity values among the three color channels, respectively, and *S* represents the low-light input image.

To improve image visibility and perceptual quality, MARN employs four loss functions to optimize the network output. The overall loss function is defined as shown in Equation 8:


$$\begin{array}{l} L_{\text{total}}=\lambda_{r}L_{r}+\lambda_{s}L_{\text{ssim}}+\lambda_{c}L_{\text{color}}+\lambda_{d}L_{\text{detail}} \end{array}$$ (8)

where λ_*r*_, λ_*s*_, λ_*c*_, and λ_*d*_ are weighting coefficients corresponding to the reconstruction loss, structural similarity loss, color loss, and detail loss, respectively. The term *L*_*r*_ denotes the reconstruction loss, *L*_*ssim*_ represents the structural similarity loss, *L*_*color*_ corresponds to the color consistency loss, and *L*_*detail*_ denotes the detail preservation loss.

Image gradients are commonly utilized for edge and texture preservation. First-order and second-order derivative operators are employed to compute gradient information. The detail loss function is expressed as shown in Equation 9:


$$\begin{array}{l} L_{detail}=||\text{Grad}\left(R_{GT}\right)-\text{Grad}\left(S*I^{-1}\right)|| \end{array}$$ (9)

where *R*_*GT*_ denotes the ground-truth reflectance image, and *Grad*(·) represents the gradient operator used to extract image details and edge information. Attention mechanism focuses on important part of image thus preserving the useful information while cleaning noise. MARN is used for low light image enhancement based on Retinex theory to improve the final image quality (Zhang and Wang, 2021).

### 2.3 Region detection and segmentation

In image processing, region detection is the process of locating and defining significant regions or areas within an image. A dataset of fetal ultrasound images along with corresponding bounding box annotations for the nuchal translucency (NT) region, are used for region localization. Segmentation is the process of dividing an image into distinct regions or segments, usually based on pixel characteristics, to facilitate analysis and tasks such as object detection and identification. A popular medical image segmentation model like U-Net is used to segment the NT region effectively (Siddique et al., 2021). U-Net has two main parts: Encoder (down sampling) and Decoder (up sampling). Encoder extracts important features like pattern and shapes to learn abstract information about the image while decoder converts the abstract information in to high resolution segmentation mask. ConvTranspose followed by batch normalization and Relu Activation is used to add non-linearity and improve the segmentation accuracy. Figure 3 shows the segmented NT regions of ultrasound scans.

**Figure 3:**
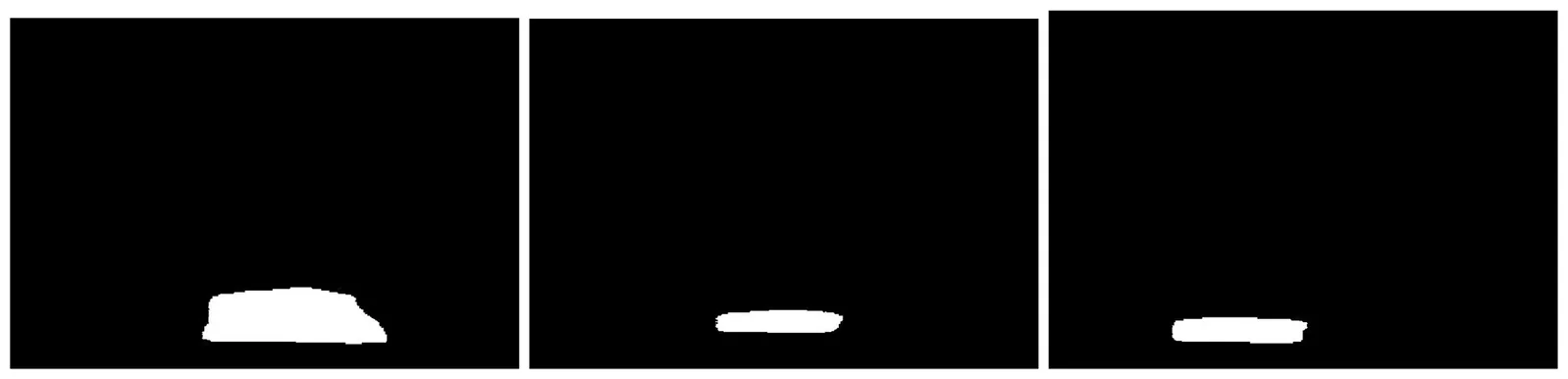
Representative NT segmentation masks generated by the U-Net model.

### 2.4 Thickness detection

The thickness detection of NT, which measures the fluid-filled region behind the fetal neck to assess the risk of chromosomal abnormalities like Down syndrome, which is also an essential part of first-trimester ultrasound screening. For this thickness detection phase, Active Contour Model can be used to measure the region boundaries by calculating based on boundary geometry. The below definitions derive the effectiveness of the thickness detection model.

#### 2.4.1 Active contour model

Active Contour Model drags outlines toward the regions to be segmented by combining statistical data from the local and global image areas (Niaz et al., 2023). The edge indicating function is further integrated with this data to precisely capture ROIs in images. Additionally, contours are forced to adhere firmly to the ROI borders by the length term based on the spf function. The edge-based ACM for local and global energy are represented as shown in Equation 10


$$\begin{array}{l} E_{\text{ELGS}}=E_{\text{ELG}}+\mu L_{t}\mathrm{extspf}\left(\phi \right) \end{array}$$ (10)

where *L*_*t*_extspf(ϕ) represents the region-based length term, μ is the width-controlling parameter, and *E*_*ELG*_ denotes the combined energy term.

The global fitting energy function can be expressed as shown in Equation 11:


$$\begin{array}{l} I_{\text{GFE}}=\nu_{1}M_{1}+\nu_{2}M_{2} \end{array}$$ (11)

where *M*_1_ and *M*_2_ denote the membership functions, while ν_1_ and ν_2_ correspond to the inner and outer regions of the contour, respectively. The term *I*_*GFE*_ represents the global fitting energy function. Once the NT thickness is estimated from the segmented region, this thickness value, along with the boundary geometric and spatial characteristics of the segmented NT region are forwarded to labeling and classification stages. NT thickness serves as the principal clinical feature for subsequent risk categorization.

### 2.5 UN-supervised based labeling

Similar NT readings are grouped together through labeling, which may indicate subgroups or patterns. These can be utilized with the technique of K-means ++ to cluster and label the data from measured thickness value in un-supervised learning approach.

#### 2.5.1 K-means++ algorithm

Randomization was utilized in k-means clustering to determine initial centroids. From the data points, the starting k-centroids were chosen at random. The issue of initialization sensitivity arises from the randomness of the selection of k-centroid sites. The final clusters that are generated are often impacted by this issue. The selection of the initial centroids determines the final clusters that can evolved. The disadvantage of k-means is addressed by K-means++, which guarantees a more intelligent initialization of the centroids and enhances the clustering quality. The algorithm is identical to the traditional k-means algorithm except for initialization. An improved clustering technique called k-means++ favors strong dissimilarity between chosen beginning centers by randomly generating initial centers. A consecutive center is chosen from the samples using a probability proportional to the distance between a sample and its closest existing center, starting with an arbitrary sample as the initial center. The K-means++ clustering generates three groups corresponding to low-, medium-, and high-risk NT observations based on the measured thickness values. These labeled observations are subsequently used in the classification stage. The measured NT thickness, together with the boundary geometry and spatial characteristics of the segmented NT region, forms the feature representation for the MSSTGAN classification stage.

### 2.6 Classification

Classification is the process of organizing data into homogeneous classes based on the shared characteristics found in the data. With the help of Multi-Scale Spatio-Temporal Graph Attention Network (MSSTGAN) to classify the chromosomal abnormalities of NT such as low, medium and high based on the thickness level of fluid. To enhance this technique, replacing the transformer attention module by transformer-based multi-head attention module, which allows the technique to similarly work with multiple, different-scaled, and potentially unrelated regions. The extracted NT information is represented as feature observations for graph-based classification after NT segmentation, thickness estimation, and labeling. Each observation is represented as a graph node, characterized by the NT thickness as the main property and including the boundary geometry and spatial characteristics of the segmented NT region. Relationships between neighboring observations define the spatial connections, while consecutive observations provide temporal information. These spatial-temporal graph representations are processed by MSSTGAN to classify NT cases into low-, medium-, and high-risk categories. The enhanced attention mechanism extends the conventional attention operation by emphasizing relevant spatial and temporal feature relationships, thereby enabling more effective representation of NT-related patterns.

#### 2.6.1 Multi-scale spatial temporal graph attention network

The MSSTGAN model is utilized for the classification of NT-based features, where inputs derived from segmented NT regions and thickness measurements are employed to predict risk levels, including low, medium, and high risk. According to the Fetal Medicine Foundation (FMF) guidelines, an NT thickness above the 95th percentile, typically ≥3.5 mm during 11–13^+6^ weeks of gestation, is considered indicative of an increased risk of chromosomal abnormalities (Elger et al., 2025). Based on this clinical threshold, the model categorizes cases into different risk groups.

The model is trained using the cross-entropy loss function and optimized with the Adam optimizer, resulting in improved classification performance through effective modeling of spatio-temporal dependencies. The MSSTGAN architecture comprises three major components: Spatio-Temporal Embedding (STE), Encoder, and Decoder. Both the encoder and decoder incorporate Pyramid Multi-Head Attention Blocks (PMAB), Transform Attention (TA), and Fully Connected (FC) layers (Tian et al., 2025). The PMAB module enables adaptive weighting of spatial and temporal features, while the STE module integrates spatial and temporal information to capture dynamic patterns within the data effectively.

In addition, the outputs of each layer are D-dimensional and residual connections can be implemented. The node representations learned from the NT feature observations are linked via spatial and temporal relationships so that the information can be aggregated from related observations. The combined neighbor information is then linearly transformed in the hidden layer to obtain the new node features in the output layer.

## 3 Result discussion

The dataset includes first-trimester ultrasound images acquired between 11 and 13 weeks + 6 days of gestation It includes 1,528 two-dimensional (2D) fetal ultrasound images from 1,519 patients, obtained from a Mendeley repository with bounding-box annotations for the NT region.

### 3.1 Inference time analysis

To evaluate the real-time applicability of the proposed framework, the inference time required to process a single ultrasound scan was measured. The measurement includes the processing stages of the proposed framework, including preprocessing, NT region detection, segmentation, NT thickness measurement, and risk classification. Model training and model initialization were excluded from the inference-time measurement because these operations are performed offline. The average inference time was calculated over the test ultrasound scans and reported in milliseconds (ms) per scan. The proposed framework required an average inference time of approximately 400 ms per ultrasound scan on the experimental hardware. This indicates that the proposed framework has potential for rapid automated NT assessment. The AUC and ROC charts for the proposed approaches are shown in Figure 4. The ROC curves were generated using the predicted class probabilities obtained from the softmax output of the classifier rather than the final hard class predictions. For each class, the false positive rate (FPR) and true positive rate (TPR) were calculated across the available probability thresholds, and the area under the ROC curve (AUROC) was calculated using the trapezoidal integration method. The obtained AUROC values were 0.983, 0.924, and 0.991 for Classes 0, 1, and 2, respectively, indicating strong discriminatory performance.

**Figure 4:**
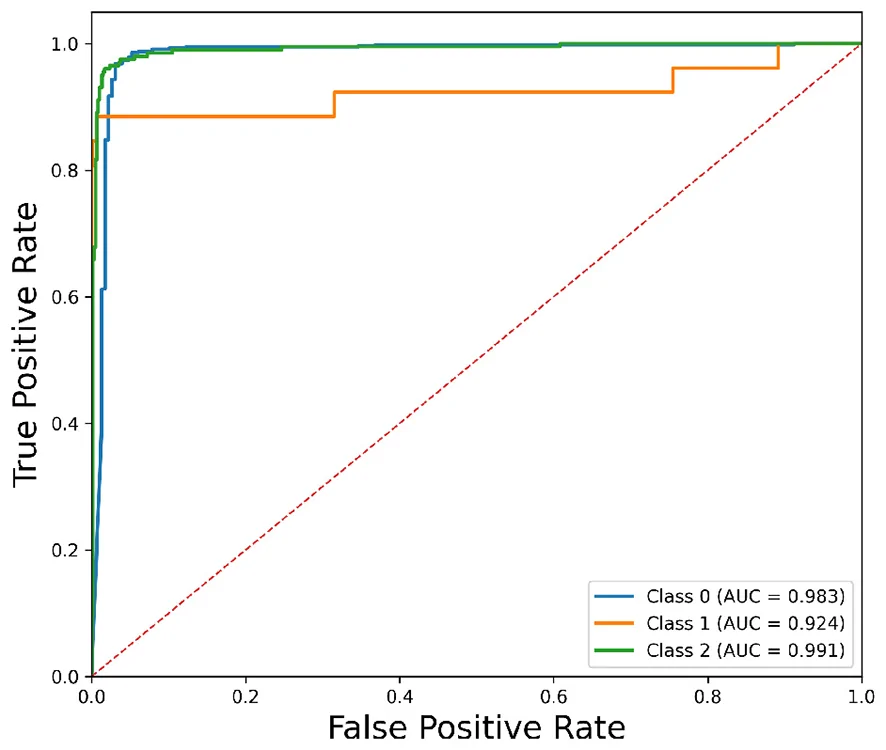
AUC and ROC plots for the proposed techniques.

Figure 5 presents the precision-recall curves for the three risk classes. The curves were generated using the predicted softmax probabilities and the corresponding true class labels. The obtained AUPRC values were 0.987, 0.883, and 0.974 for Classes 0, 1, and 2, respectively. The curves demonstrate high precision over a broad range of recall values, with a decline in precision at very high recall levels due to the inclusion of additional false-positive predictions.

**Figure 5:**
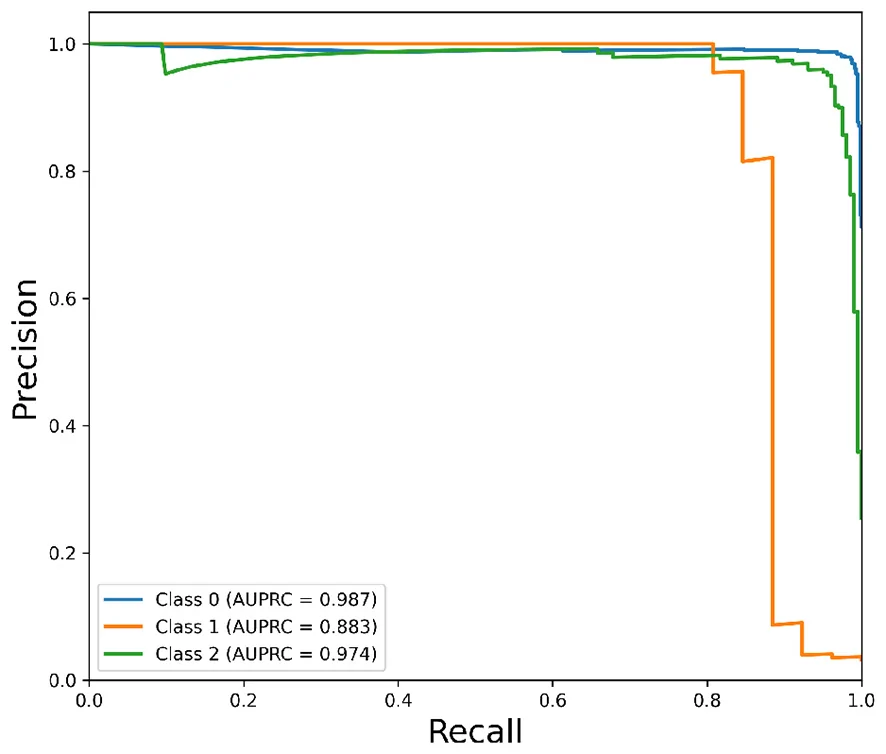
Precision-recall curve for classifier model.

Figure 6 shows the comparison between predefined probabilities and actual outcomes using calibration curve. The model's projected probability and actual result frequencies are completely calibrated, as shown by the dotted line.

**Figure 6:**
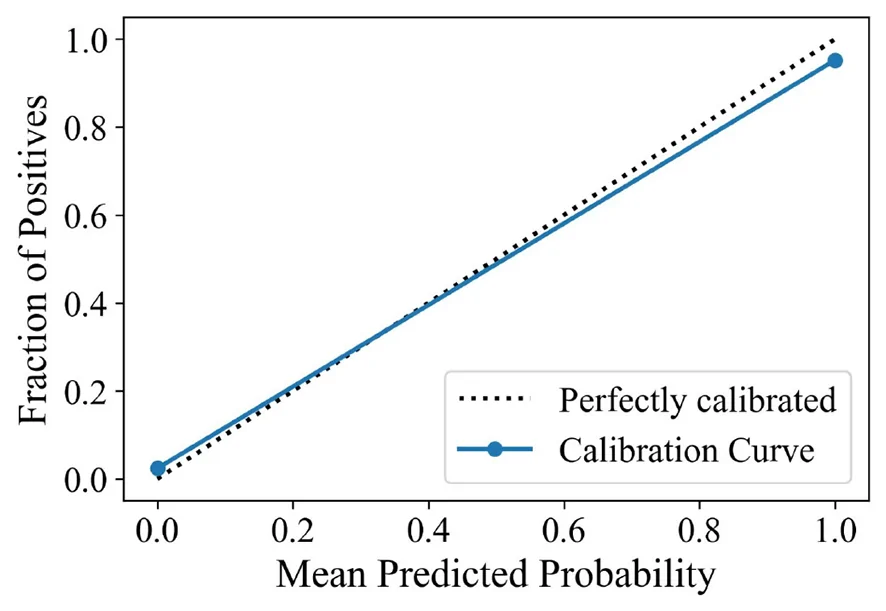
Assessment of calibration curve.

### 3.2 Comparison analysis for classification

To evaluate the performance of the proposed EMSSTGAN model, it is compared with some existing algorithms such as Artificial Neural Network-Machine Learning (ANN-ML) (Chen et al., 2025), Spatio-Temporal Convolutional Neural Network (2D+t-CNN) and Deep Convolutional Neural Network (DCNN). To provide for a fair comparison with the proposed EMSSTGAN model, all baseline models are created and evaluated individually using the same dataset, training/testing split and experimental conditions. Hence, the stated performance numbers for the baseline models are obtained in our experiments and not directly taken from the respective published articles.

Figure 7 presents the accuracy and precision comparison of EMSSTGAN with existing methods. The proposed EMSSTGAN classifier attains an accuracy of 98.15% and a precision of 97.56%, demonstrating superior classification performance and improved reliability over the benchmark models. Comparison of Recall, Specificity, F1 score, and NPV is shown in Figure 8. The proposed EMSSTGAN model has a Recall value of 91.63% Specificity value of 97.77%, F1 score 94.5% and NPV value of 98.09% which is comparatively high as compared to other existing methods.

**Figure 7:**
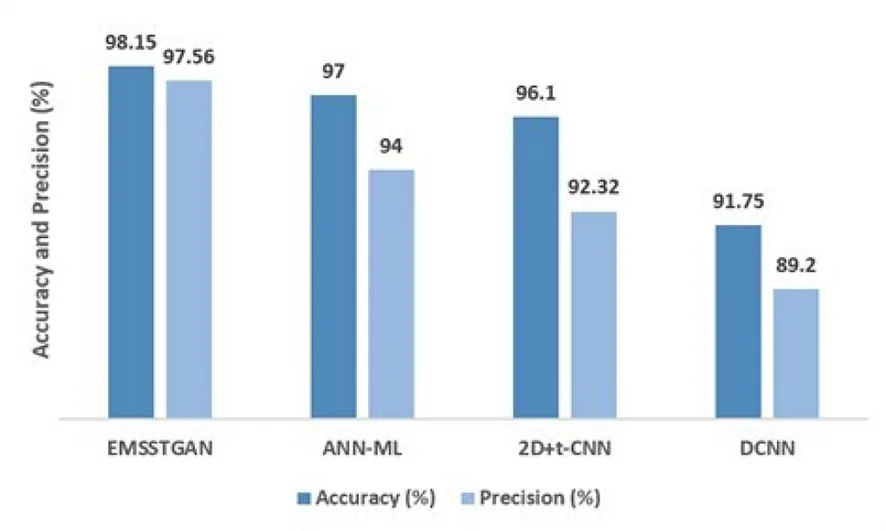
Comparison of EMSSTGAN accuracy and precision among different algorithms.

**Figure 8:**
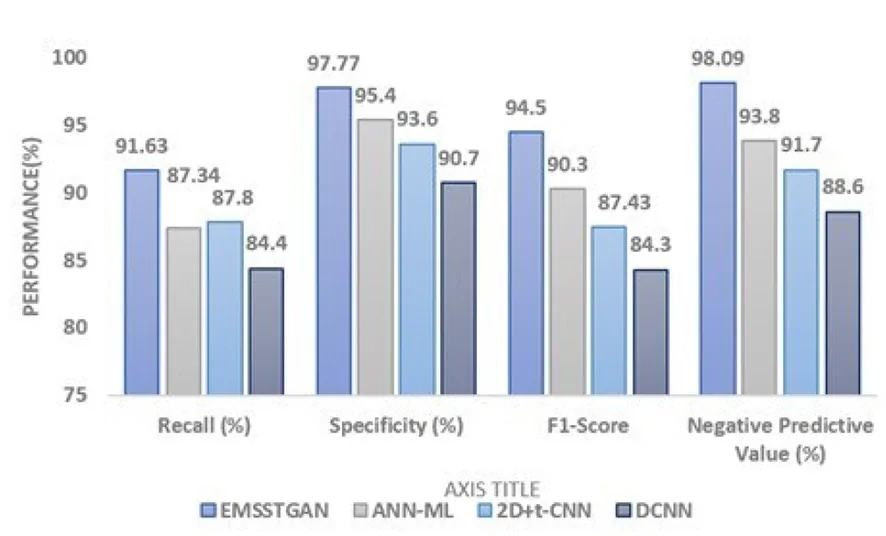
Performance evaluation of EMSSTGAN and existing methods.

### 3.3 Comparison analysis for error based performance metrics

Table 1 presents the error-based performance metrics that provides a comprehensive assessment of the reliability of the proposed EMSSTGAN classification model in comparison with the other traditional models. Lower values of these metrics indicate fewer misclassifications, leading to more accurate and dependable prenatal risk assessment.

**Table 1:** Comparison of error-based performance metrics.

Method	FNR (%)	Error (%)	FPR (%)	FOR (%)	FDR (%)
EMSSTGAN	8.37	1.85	2.23	1.91	2.44
ANN-ML	12.66	3.00	4.60	6.20	6.00
2D+t-CNN	12.20	3.90	6.40	8.30	7.68
DCNN	15.60	8.25	9.30	11.46	10.80

## 4 Conclusion

NT is a prenatal ultrasound that is done during the first trimester. It aids in assessing the likelihood that a fetus may have congenital disorders, such as Down syndrome. As the cornerstone of prenatal screening, NT measurements might be difficult to carry out. These difficulties include differences in imaging settings that might produce abnormalities in the data and operator reliance, which mostly depends on the technician's ability. To tackle these issues, an EMSSTGAN model was proposed to detect the risk of NT. The input image of NT is collected and preprocessed using the FRANet technique to remove unwanted noise and the MARN technique to enhance the contrast level of the image. After region detection, the images are segmented using the U-Net technique to isolate the NT region within the bounding box. The labeled data can then be classified using EMSSTGAN to classify the risk of chromosomal abnormalities in the NT region. The proposed approach outperforms the accuracy of 98.15%, Precision of 97.56% and Recall of 91.63%, respectively. Compared to other existing methods, these results demonstrate that EMSSTGAN offers more advantages.

## 5 Future scope

The proposed techniques require large datasets to learn effectively. Validation in real-world clinical settings is still an important challenge. Insufficient data may lead to overfitting and reduced generalization. An explicit ablation study was not conducted to independently quantify the contributions of FRANet, MARN, U-Net, ACM, and the modified attention mechanism; therefore, the reported results reflect the performance of the integrated framework. Future work will include systematic component-wise ablation experiments and explore the use of specific LLMs to generate interpretable clinical reports and patient-friendly counseling summaries from the outputs of the proposed framework, improving the transparency and accessibility of AI-supported prenatal risk assessment (Lugowski et al., 2025).

## Data Availability

Publicly available datasets were analyzed in this study. This data can be found here: Dataset for Fetus Framework, Mendeley Data, doi: 10.17632/n2rbrb9t4f.1.
